# β-arrestin and myosin VI cooperatively control glucose-dependent insulinotropic peptide receptor (GIPR) internalization and signaling dynamics

**DOI:** 10.1016/j.jbc.2026.113339

**Published:** 2026-07-16

**Authors:** Nishaben M. Patel, Sivaraj Sivaramakrishnan

**Affiliations:** Department of Genetics, Cell Biology and Development, University of Minnesota, Minneapolis, Minnesota, USA

**Keywords:** cell imaging, GPCR, incretin, signaling, trafficking

## Abstract

Glucose-dependent insulinotropic peptide receptor (GIPR) stimulates insulin release and regulates metabolic homeostasis. GIPR function is shaped by spatiotemporal trafficking of this G protein-coupled receptor (GPCR). While GPCR endocytosis is traditionally associated with β-arrestin, GIPR internalization is only modestly dependent on this pathway. In this study, we demonstrate that GIPR engages a cytoskeletal motor, myosin VI to drive receptor endocytosis. GIPR engages the adaptor-motor complex through a PDZ-binding motif (PBM) at its C-ail. Interestingly, β-arrestin binding to phosphorylated residues upstream of the PBM enhance myosin VI recruitment and activation. GIPR internalization is dependent on both receptor phosphorylation and the PBM site to recruit β-arrestin and myosin VI, respectively. Cooperative engagement of β-arrestin and myosin VI results in desensitization of GIP-stimulated cAMP signaling while activating pERK1/2 from endosomal compartments. Blocking myosin VI activity enhances insulin release in pancreatic beta cells, demonstrating a novel role for this pathway in regulating the physiological effects of GIPR. Our findings highlight the direct convergence of two independent trafficking pathways at the level of the receptor C-tail, with implications for the nuanced regulation of individual GPCRs through the differential engagement of β-arrestin and myosin VI.

G protein-coupled receptor (GPCR) internalization is an established mechanism for receptor desensitization, compartmentalized signaling, and spatiotemporal regulation of physiological responses (1, 2). Clathrin-dependent GPCR endocytosis typically proceeds through the recruitment of β-arrestin to a barcode of GRK phosphorylated residues in the GPCR C-tail (3). In parallel, ∼ 30% of GPCR C-tails contain a PDZ-binding motif (PBM) that can recruit PDZ adaptor proteins to facilitate receptor trafficking (4, 5, 6, 7). The relative effects of β-arrestin and PDZ adaptors on GPCR internalization is dependent on the specific sequence motifs in the receptor C-tail (8, 9). The PBM is typically located in the last 3 to 5 amino acids of the receptor C-tail (8). In contrast, GRK phosphorylation sites are dispersed throughout the C-tail and their differential phosphorylation mediates distinct downstream signaling effects through conformational effects on β-arrestin (9). While these two pathways have been extensively studied, their potential intersection in regulating signaling and physiological responses in the same receptor is unclear and forms the premise of this study.

This study focuses on glucose-dependent insulinotropic peptide receptor (GIPR), whose differential desensitization is of interest in targeting metabolic disorders (10). GIPR is a class B GPCR that regulates glucose metabolism, body weight, bone formation and food intake (11, 12). GIPR, along with its counterpart GLP1R, is responsible for the increase in insulin secretion from beta cells in the islets of Langerhans postprandially (13). This process is referred to as the incretin effect and is compromised in type 2 diabetes (T2D) (14). Incretin receptors are prime targets of weight loss and T2D medications (12). Incretin mimetics have transformed the medical landscape for treatment of obesity and T2D. Both agonism and antagonism at GIPR have been reported to have positive effects on body weight (10). Chronic agonism of GIPR desensitizes the cAMP signaling response of the receptor (15). Additionally, co-agonism at GIPR and GLP1R has achieved superior weight loss results, compared to mono-agonism at GLP1R (12, 16). These contrasting results highlight the gap in our knowledge of GIPR function and regulation, suggesting the need for further insights into GIPR trafficking to reconcile previous findings.

Membrane trafficking of GIPR is an essential factor in shaping the signaling and physiological response of this receptor (17). GIPR is constitutively internalized and recycled through the fast-recycling pathway (18). Binding of GIP reroutes the receptor to the trans Golgi network (TGN) and the slow-recycling pathway (18). The molecular mechanisms that govern GIPR trafficking are relatively unexplored. Additionally, the pathways that govern differential signaling of therapeutic drugs, compared to the native agonist, are unknown. Further insights into the regulatory pathways of GIPR that guide receptor trafficking and recycling, desensitization, and drug tolerance are required to delineate mechanisms of action of medications for obesity and T2D.

GIPR has an extensive C-tail with multiple phosphorylation sites that recruit β-arrestin, and a putative PBM (8, 19). We have demonstrated previously that GPCRs can recruit myosin VI through the PBM site (20). Hence, in this study, we use GIPR to delineate the intersection between two trafficking pathways - β-arrestin and myosin VI. We demonstrate that β-arrestin and myosin VI activity are both required for GIPR internalization, cAMP production and phosphorylation of ERK. β-arrestin enhances binding of GIPC, an adaptor of myosin VI, to the phosphomimetic GIPR C-tail, stimulating motor activity. Finally, we show that myosin VI-dependent internalization desensitizes GIPR-mediated insulin secretion in pancreatic beta cells. Together, our data support a model wherein GIPR internalization is cooperatively regulated by β-arrestin and myosin VI, leading to desensitization of insulin secretion.

## Results

### GIPR internalization requires both β-arrestin and myosin VI

**Figure 1:**
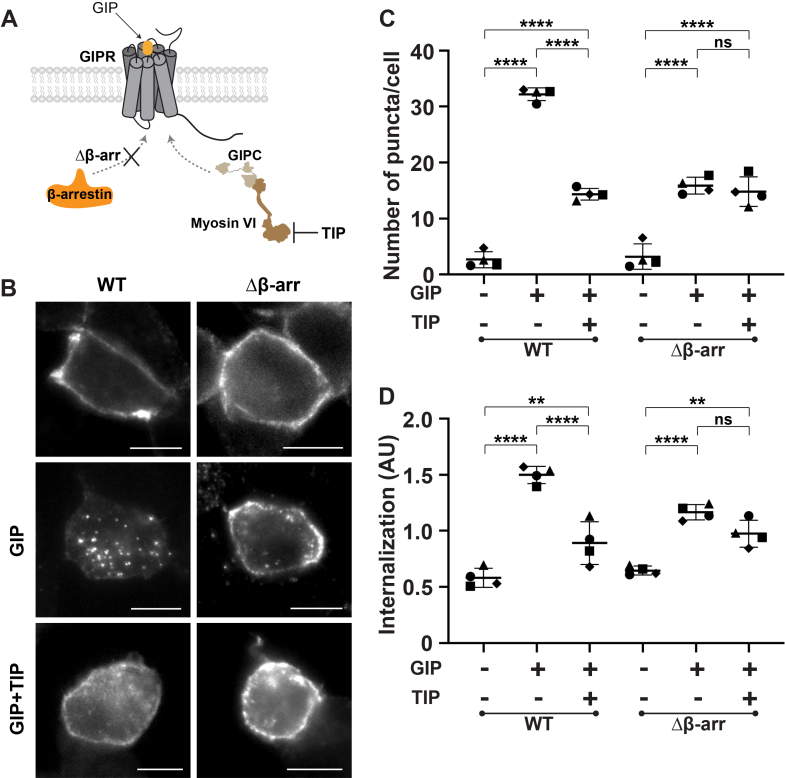
**Myosin VI and β-arrestin dually regulate GIPR internalization.***A*, schematic representation of GIPR regulation by myosin VI and β-arrestin. GIPR C-tail has a PDZ-binding motif (PBM) that binds PDZ adaptor GIPC, which in turn recruits cytoskeletal motor myosin VI. GIPR also recruits β-arrestin through multiple phosphorylation sites in its C-tail. Δβ-arr indicates β-arrestin half knockdown in HEK293 cells. TIP is a chemical inhibitor of myosin VI activity. *B*, internalization of GIPR with N-terminal FLAG tag, visualized using anti-FLAG antibody and secondary AlexaFlour 546. HEK293 wild-type (WT) and Δβ-arr cells expressing GIPR were stimulated with GIP (1 μM) for 15 min at 37 °C. Cells were treated with TIP (100 μM) to block myosin VI activity. Scale bar, 10 μm. *C* and *D*, number of puncta/cell (*C*) and cytosol-to-cell surface intensity (*D*) for GIPR internalization in HEK293 WT or Δβ-arr cells under basal, GIP or GIP + TIP conditions. Data is represented as mean ± SD for four biological replicates. One-way ANOVA with Tukey’s *post hoc* test, ∗∗∗∗*p* < 0.0001, ∗∗*p* < 0.01.

**Figure 2:**
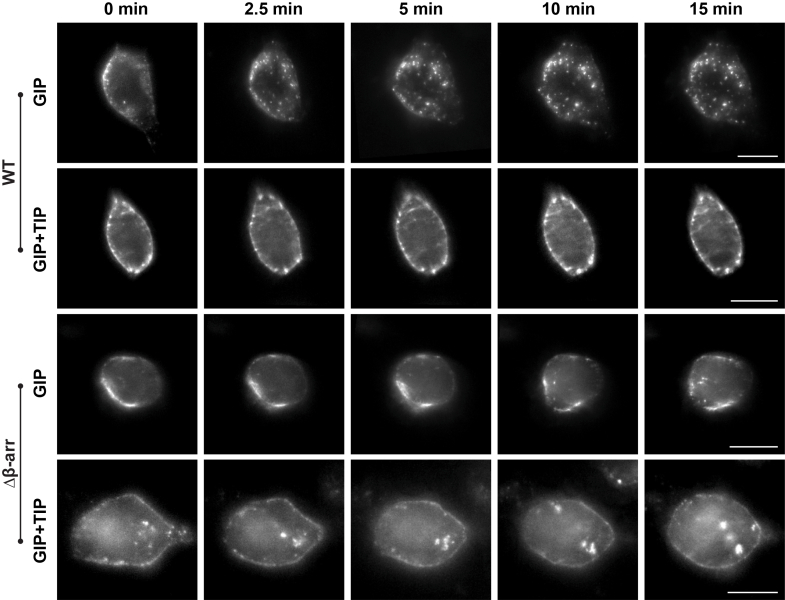
**Time-course of GIPR internalization.** Live-cell imaging of GIPR in HEK293 WT or Δβ-arr cells. FLAG-tagged GIPR was labeled with anti-FLAG antibody and secondary AlexaFlour 546. Cells were stimulated with GIP ± TIP. Scale bar, 10 μm.

GIP stimulation of GIPR has been shown to enhance receptor internalization in a β-arrestin2 dependent manner (18). GIPR has a type III PDZ-binding motif (X-X-C) that we have recently shown to engage a cytoskeletal trafficking pathway in the D2 dopamine receptor (D2R) (8, 20). To examine the relative roles of these two mechanisms, we used a myosin VI-selective inhibitor (TIP) and a β-arrestin dual-knockdown cell line (Δβ-arr) (Fig. 1*A*) (21, 22). GIP treatment (1 μM; 15 min) stimulated GIPR internalization, as visualized using antibody-labeled GIPR in HEK293 cells (Fig. 1*B*). Consistent with previous studies, constitutive knockdown of β-arrestin (Δβ-arr) yields reduced internalization (Fig. 1*B*), as quantified from the number of internalized puncta per cell (Fig. 1*C*) and cytosol-to-surface ratio of fluorescence intensity (Fig. 1*D*) (23). TIP pretreatment (100 μM; 15 min) also abolishes GIPR internalization (Fig. 1, *B–D*) with no further effects observed with both Δβ-arr and TIP treatment. Next, we performed live-cell imaging to track GIPR internalization over time. In HEK293 WT cells, GIPR completely internalizes to endosomal compartments within 15 min of agonist treatment (Fig. 2). In contrast, TIP treatment or β-arrestin knockdown reduces GIPR internalization (Fig. 2). These data demonstrate that both β-arrestin and myosin VI activity are necessary for GIP-stimulated GIPR internalization.

**Figure 3:**
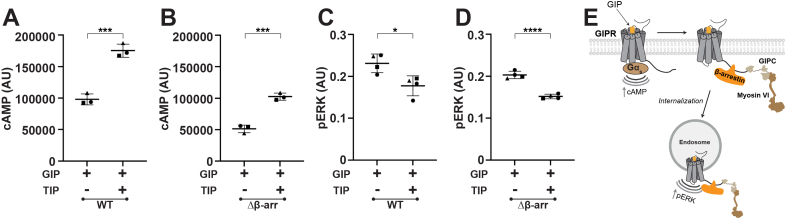
**Myosin VI and β-arrestin regulate GIPR signaling.***A* and *B*, cAMP levels in HEK293 WT (*A*) or Δβ-arr cells (*B*) expressing GIPR and stimulated with GIP (1 μM) ± TIP (10 μM) for 15 min at room temperature. *C* and *D*, pERK signaling for GIPR expressed in HEK293 WT (*C*) or Δβ-arr (*D*), in response to GIP (1 μM) ± TIP (10 μM) for 15 min at room temperature. Mean ± SD for at least three biological replicates. Unpaired *t* test, ∗∗∗∗*p* < 0.001, ∗∗∗*p* < 0.001, ∗*p* < 0.05. *E*, schematic representation for regulation of GIPR signaling by myosin VI and β-arrestin. Inhibition of myosin VI results in elevated G_s_ signaling (cAMP production) from the plasma membrane. MAPK signaling (pERK) from endosomal compartments decreases with ablation of myosin VI activity or knockdown of β-arrestin.

GIP-stimulated internalization has been shown to result in reduced cAMP signaling from the remaining cell surface receptors (24). Accordingly, blocking GIPR internalization through TIP treatment (10 μM; 15 min) in HEK293 cells with transient GIPR overexpression, significantly enhances cAMP accumulation in response to GIP stimulation (1 μM; 15 min) (Fig. 3*A*). Interestingly, these augmented cAMP levels with TIP treatment are observed both in WT and Δβ-arr cells (Figs. 3, *A* and *B* and S1*A*). Despite matched receptor expression and cell counts, GIP-stimulated cAMP levels are lower in Δβ-arr cells compared to their WT counterparts. This observation is not unique to GIPR, as WT and Δβ-arr cells expressing β2AR, a prototypical Gs-coupled receptor, retain this differential response (Fig. S2). Nonetheless, in both WT and Δβ-arr cells, blocking myosin VI activity does enhance cAMP. Hence, we conclude that blocking receptor endocytosis through the myosin VI pathway limits desensitization of cAMP signaling.

GIP stimulation is known to trigger ERK1/2 phosphorylation with a peak response at 4 min (25). We observe pERK1/2 stimulation in HEK293 cells with transient GIPR overexpression both in WT and Δβ-arr cells (Figs. 3, *C* and *D* and S3). In both cell types, TIP treatment significantly diminishes pERK1/2 levels (Figs. 3, *C* and *D*, S1*B*, S3). Given the lack of internalization upon TIP treatment (Figs. 1, *B–D*, 2), we infer that MAPK activity stems from internalized receptor (Figs. 3*E* and S3).

While receptor internalization measurements were conducted at 37 °C (Figs. 1, 2), cAMP and pERK assays were performed at room temperature to adapt them to a standard plate-reader format (Fig. 3) (22, 26). Given that temperature is known to affect the rate of endocytosis, we performed matched signaling assays at both room temperature and 37 °C. We find that the TIP-dependent increase in cAMP levels and reduction in pERK response is preserved at 37 °C (Fig. S4).

### GIPC is recruited to GIPR prior to agonist treatment

**Figure 4:**
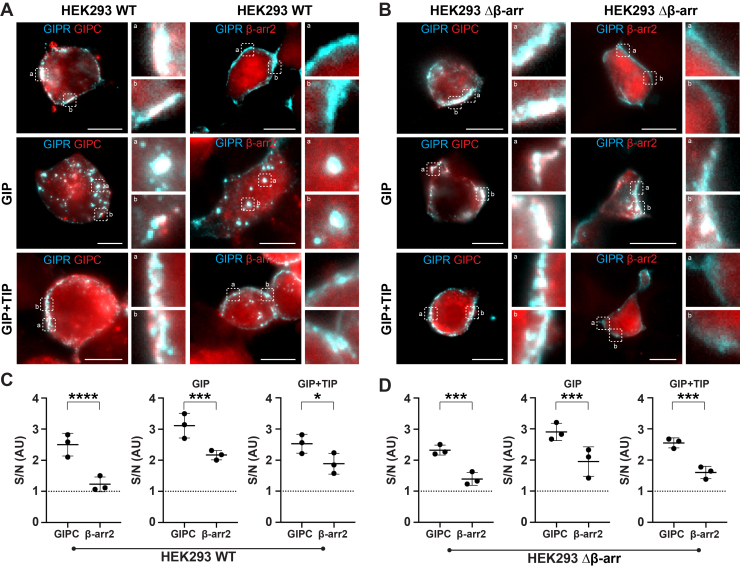
**GIPC recruitment to GIPR is independent of β-arrestin.***A* and *B*, merged images for colocalization of GIPR with GIPC or β-arr2 in HEK293 WT (*A*) or Δβ-arr cells (*B*), under basal condition or GIP stimulation ± TIP for 15 min at 37 °C. HEK293 cells expressing FLAG-GIPR with GIPC or β-arr2 were visualized by immunostaining the receptor with anti-FLAG antibody and secondary AlexaFlour 647. Insets display magnified view of the areas marked with dashed squares. Scale bar, 10 μm. Refer to Figure. S5 for images of the individual channels. *C* and *D*, colocalization analysis of GIPR to GIPC or β-arr2 in HEK293 WT (S) and Δβ-arr (*D*) cells under basal condition or GIP ± TIP stimulation. Signal to noise ratio above 1 indicates colocalization. Mean ± SD for at least three biological replicates. Two-way ANOVA with Sidak’s *post hoc* test, ∗∗∗∗*p* < 0.001, ∗∗∗*p* < 0.001, ∗*p* < 0.05.

We have previously shown that a PDZ adaptor protein, GIPC, recruits myosin VI to PBMs in GPCR C-tails (20). Hence, we examined GIPC localization in cells expressing GIPR. GIPC-TagRFP fusions co-expressed with N-terminal FLAG-tagged GIPR in both WT and Δβ-arr HEK293 cells, show co-localization of Tag-RFP and Alexa Fluor 647 (M2 FLAG monoclonal + fluorescent secondary) at the cell plasma membrane prior to GIP treatment (Figs. 4 and S5). Agonist (GIP; 1 μM; 15 min) stimulation results in receptor internalization, with persistent GIPC colocalization observed in endomembrane compartments in WT cells. While β-arrestin knockdown and TIP pretreatment both result in substantially lower internalization, GIPC colocalization persists both at the plasma membrane and endomembrane compartments (Fig. 4). Dual inhibition of myosin VI and β-arrestin abolishes GIP-stimulated internalization, resulting in GIPC colocalizing with GIPR at the plasma membrane. Together, these data demonstrate that GIPC is recruited to the receptor independent of agonist, β-arrestin, or myosin VI activation.

In contrast, β-arrestin does not localize to GIPR in the absence of GIP treatment (Figs. 4 and S5). TagRFP-β-arrestin2 (β-arr2) fusions remain cytosolic when co-expressed with FLAG-tagged GIPR (visualized with M2 FLAG antibody mixed with Alexa Fluor 647 secondary). GIP stimulation results in β-arrestin recruitment to GIPR endosomes. GIP stimulation with dual inhibition of myosin VI and β-arrestin mimics unstimulated cells, with cytosolic β-arrestin and receptor at the plasma membrane. We conclude that consistent with its established mode of interaction (27), β-arrestin is only recruited to the receptor following GIP stimulation, with visible colocalization with GIPR at endosomal compartments.

Interestingly, we observed that β-arrestin translocates to the nucleus, despite the presence of a nuclear exclusion sequence in β-arrestin (Fig. 4) (28). However, we find that β-arrestin does not localize to the nucleus when expressed alone or co-expressed with the vasopressin 2 receptor (V2R) (Fig. S6). We conclude that GIPR co-expression drives β-arrestin localization to the nucleus, in an agonist-independent manner.

### β-arrestin and GIPC interactions with GIPR are both essential for receptor internalization

**Figure 5:**
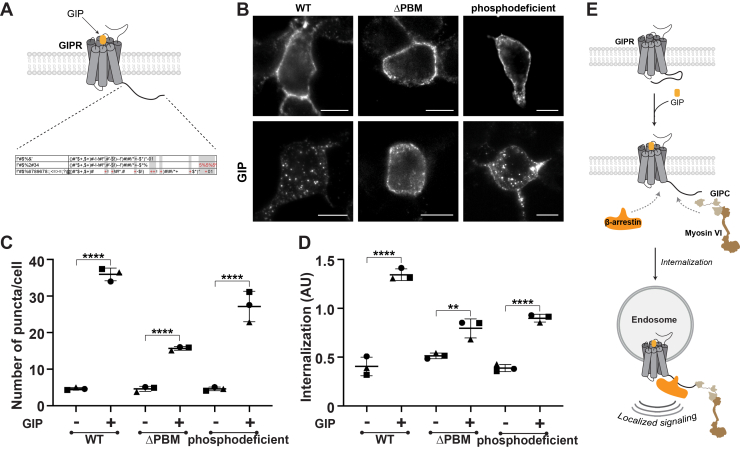
**GIPR internalization is dependent on PBM sequence and phosphorylation in the C-tail.***A*, schematic representation of GIPR variants used in internalization assay. GIPR ΔPBM was created by removing the last five residues of the C-tail. Phosphorylation sites highlighted in gray were mutated to alanine to generate GIPR phosphodeficient construct. *B*, representative images of HEK293 WT cells expressing GIPR WT, ΔPBM and phosphodeficient, under basal and GIP (1 μM) stimulation at 37 °C. Refer Figure. S7 for GIPR phosphomimetic data. For surface labeling of the receptor, cells were incubated with anti-FLAG antibody and AlexaFlour 546/647. Scale bar, 10 μm. *C* and *D*, number of puncta/cell (*C*) and ratio for cytosol-to-cell membrane intensity (*D*) for GIPR WT, ΔPBM, phosphodeficient under basal and GIP-stimulated cells. Data is represented as mean ± SD for three biological replicates. One-way ANOVA with Tukey’s *post hoc* test. ∗∗∗∗*p* < 0.0001, ∗∗*p* < 0.01. *E*, model schematic for regulation of GIPR internalization and signaling by cooperative engagement of GIPC-myosin VI and β-arrestin.

Given the colocalization of GIPC and β-arrestin with GIPR on endosomes, we examined the significance of their interactions with the GIPR C-tail for receptor internalization. The GIPC PBM site was abolished by truncating the last five amino acids (ΔPBM - LESYC), whereas a phosphodeficient construct was used to limit β-arrestin engagement (Fig. 5*A*). The phosphodeficient construct was generated by mutagenizing residues in the GIPR C-tail that were previously identified as sites phosphorylated in the activated receptor (19). Removing the PBM motif substantially reduced GIPR internalization (Fig. 5*B*), with reduced punctae per cell (Fig. 5*C*) and cytosol-to-membrane fluorescence ratio (Fig. 5*D*). The phosphodeficient mutant also demonstrated lower GIPR internalization (Fig. 5, *B–D*), albeit to a lesser extent than the PBM truncation. We also observe similar levels of internalization for the phosphomimetic variant of GIPR, compared to the wild-type receptor (Fig. S7). These data further substantiate the dual role of β-arrestin and GIPC interactions in GIPR internalization.

### GIPC engagement of GIPR C-tail is enhanced by β-arrestin

**Figure 6:**
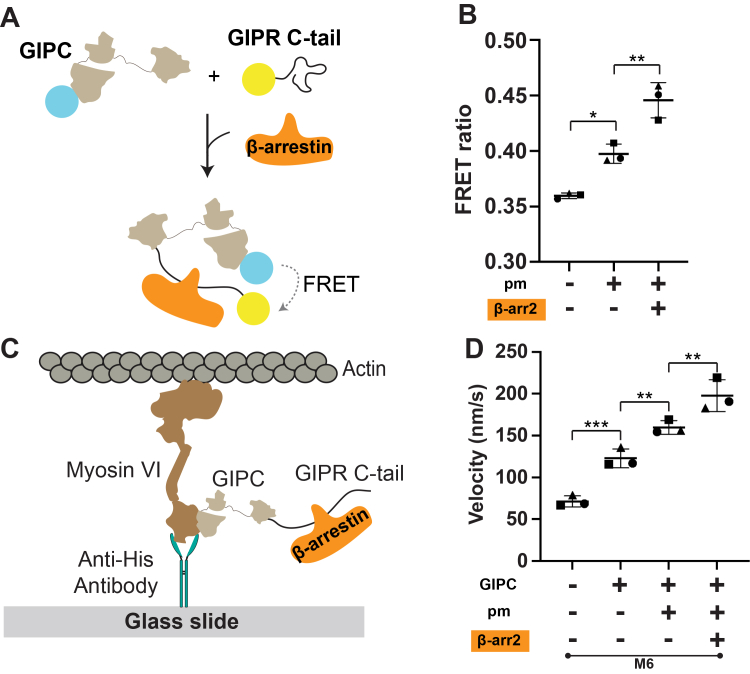
**β-arrestin enhances GIPC engagement of the GIPR C-tail and myosin VI activity.***A*, schematic of bimolecular FRET experiment between mCer-GIPC and mCit-GIPR C-tail. Wild-type (wt), phosphodeficient (pd), and phosphomimetic (pm) versions of the GIPR C-tail were used (Fig. S9*A*). Purified β-arrestin (β-arr2) was included with GIPR pm condition. (*B*) FRET ratio (525/475 nm) for bimolecular FRET between mCer-GIPC (30 nM) and mCit-GIPR C-tail pm (300 nM) ± β-arr2 (300 nM). Data is represented as mean ± SD for three biological replicates. One-way ANOVA with Tukey’s *post hoc* test. ∗∗*p* < 0.01, ∗*p* < 0.05. *C*, schematic for surface motility assay to determine myosin VI velocity. *D*, myosin VI speed with GIPC (2 μM), GIPR C-tail pm (100 μM) and β-arr2 (1 μM). Data is represented as mean ± SD for three biological replicates. One-way ANOVA with Tukey’s *post hoc* test. ∗∗∗*p* < 0.001, ∗∗*p* < 0.01. Refer to Fig. S9 for FRET and motility data of GIPR C-tail WT and pn.

We used an *in vitro* FRET-based assay to further examine interactions between GIPC, β-arrestin and GIPR C-tail (Fig. 6*A*). Recombinant mCerulean-tagged GIPC (donor) demonstrates significant FRET (quantified using FRET ratio) with mCitrine-tagged GIPR C-tail (Figs. 6*B* and S8). Both phosphomimetic (pm) and phosphodeficient (pd) versions of the recombinant GIPR C-tail show similar FRET ratios, supporting GIPC interactions with GIPR C-tail prior to agonist stimulation (Fig. S9*B* and Table S1). Addition of recombinant β-arrestin enhances this interaction, suggesting that GIPC and β-arrestin cooperatively bind the GIPR C-tail. To examine the functional consequences of this cooperativity, we used an established *in vitro* motility assay that reports on myosin VI activity as witnessed by the speed of actin filaments gliding on a myosin coated surface (Fig. 6*C*). As previously reported, GIPC stimulates actin gliding speeds (20, 29), with an increase observed upon addition of a phosphomimetic GIPR C-tail peptide (Figs. 6*D*, S9*C* and Table S2). Addition of recombinant β-arrestin further augments actin gliding speed (Fig. 6*D*). Together, these data demonstrate cooperativity between GIPC and β-arrestin in stimulating myosin VI activity through engagement of the GIPR C-tail.

### Myosin VI mediates insulin secretion at GIPR

**Figure 7:**
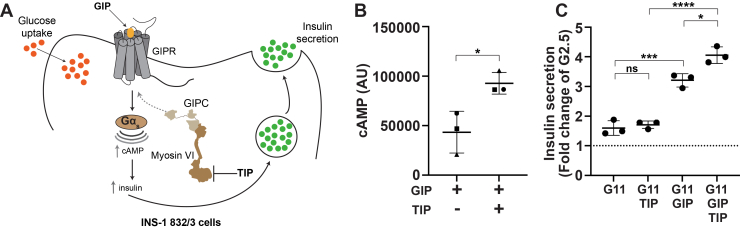
**Myosin VI desensitizes incretin signaling at GIPR.***A*, schematic depiction of glucose-stimulated insulin secretion at GIPR. In the presence of glucose, GIP stimulation of GIPR leads to cAMP production, resulting in insulin secretion from pancreatic beta cells. *B*, cAMP response in INS cells, under GIP stimulation ± TIP for 15 min. Unpaired *t* test. ∗*p* < 0.05. *C*, glucose-dependent insulin secretion by INS cells, in response to GIP stimulation ± TIP for 30 min at 2.5 mM (G2.5) or 11 mM glucose (G11) at 37 °C. Data represented as fold-increase over the respective 2.5 mM glucose condition. Mean ± SD for three biological replicates. One-way ANOVA with Tukey’s *post hoc* test. ∗∗∗∗*p* < 0.0001, ∗∗∗*p* < 0.001.

Previous studies have shown that β-arrestin is necessary for enhanced insulin release in response to GIP stimulation in pancreatic islets (30, 31, 32). Our data show that β-arrestin augments GIPC recruitment to the GIPR C-tail upstream of myosin VI activation. Hence, we tested whether myosin VI activity plays a role in GIP stimulated insulin secretion using a rat insulinoma cell line (INS-1832/3) (Fig. 7*A*). TIP treatment (10 μM) enhanced GIP-stimulated (1 μM) cAMP release (Fig. 7*B*) and insulin secretion under high glucose conditions (Figs. 7*C* and S10), supporting a role for myosin VI in desensitization of physiological responses of GIPR.

## Discussion

GIPR signaling is essential for glucose-stimulated insulin release from pancreatic beta cells, making it a prominent target in metabolic disorders. Insulin response to the native agonist, GIP is influenced by the spatiotemporal trafficking of this receptor. Unlike prototypical GPCRs (3), β-arrestin knockdown has previously been shown to result in only modest effects on GIPR internalization (23). We address this knowledge gap by identifying a novel incretin receptor trafficking mechanism through the engagement of the cytoskeletal motor, myosin VI. β-arrestin binds GIPR through established phosphorylation sites in the receptor C-tail (19). We find that β-arrestin binding augments GIPR C-tail interactions with a PDZ adaptor GIPC (Fig. 6*B*). GIPC in turn recruits and activates the cytoskeletal motor myosin VI to promote GIPR endocytosis (Fig. 6*D*). Blocking myosin VI activity promotes GIPR retention at the plasma membrane (Figs. 1, *B–D* and 2) with consequent enhanced cAMP signaling and reduced pERK1/2 activation from endosomal compartments (Fig. 3). β-arrestin knockdown has been previously demonstrated to enhance GIP-stimulated insulin release in pancreatic islets (32). Here, we find that myosin VI activity promotes desensitization of the insulin response in rat insulinoma cells, demonstrating the significance of this pathway in incretin receptor physiology (Fig. 7).

Our study highlights the direct intersection between two trafficking pathways at the level of the receptor. We observe that GIPC, the adaptor for myosin VI, co-localizes with GIPR, irrespective of agonist stimulation (Fig. 4). However, β-arrestin colocalization with GIPR is dependent on agonist treatment (Fig. 4) (27). This suggests that although both pathways are important for GIPR internalization, GIPC-myosin VI engagement with GIPR likely occurs prior to β-arrestin recruitment. Nonetheless, binding of β-arrestin further strengthens the interactions between GIPC-myosin VI and GIPR, as evident in our results from binding and motility assays (Fig. 6, *B* and *D*). Hence, our findings demonstrate that these two trafficking pathways, rather than being mutually exclusive, operate cooperatively to drive GIPR internalization.

While myosin VI inhibition enhances cAMP signaling in both WT and Δβ-arr cells, we find that β-arrestin knockdown attenuates GIP-stimulated cAMP response (Fig. 3*A*). Our observations contrast with previous reports that demonstrate enhanced cAMP signaling in the absence of β-arrestin (33, 34, 35). We speculate that the diminished cAMP response in Δβ-arr cells, relative to WT, could be attributed to differences in receptor reserve, PDE activity, or indirect effects of chronic β-arrestin loss. Nonetheless, our data support a significant role for myosin VI in GIPR signaling in the presence or absence of β-arrestin (Figure 3, Figure 7). Further, our colocalization experiments reveal an interesting trait of β-arrestin. While β-arrestin is absent from the nucleus when expressed alone (Fig. S6), GIPR co-expression results in the nuclear translocation of β-arrestin (Figs. 4 and S5). This property is GIPR-dependent, as we do not observe nuclear translocation for the prototypical β-arrestin-dependent receptor V2R. A previous study reported a similar phenomenon, wherein activation of the odorant receptor hOR17-4 results in localization of β-arrestin to the nucleus (36).

The GPCR C-tail is an intrinsically disordered region (IDR) that autoregulates receptor signaling and engages effectors (β-arrestin/PDZ adaptors) to influence trafficking (37). We have limited structure-function insight into the C-tail of GPCRs, given the inherent conformational heterogeneity of IDRs. Their structural plasticity allows IDRs to engage with interacting partners through versatile and atypical mechanisms (38). Recent studies have highlighted the role of GPCR IDRs in the autoregulation of receptor activity by masking G protein access to the cytosolic cavity (26, 39). Heng *et al*. revealed that the β2AR C-tail interacts with intracellular loops (ICLs) to autoinhibit the receptor under basal conditions and block access to Gs (39). We have previously demonstrated that intramolecular interactions release spatial distance constraints in β2AR ICL3, facilitating Gs coupling (26). Further, biophysical studies demonstrate changes in GPCR C-tail conformation upon phosphorylation or effector binding, with unknown functional consequences (40, 41). Our data suggest cooperative engagement of β-arrestin and GIPC with the GIPR C-tail (Fig. 6*B*). We speculate that binding of one effector reduces conformational entropy of the C-tail, increasing access to another interacting partner.

GIPR represents a receptor that engages both β-arrestin and GIPC through cooperative interactions. Individually, GPCRs can differentially engage both PDZ adpators and β-arrestin. Receptors either weakly (class A) or robustly (class B) recruit β-arrestin depending on the number and pattern of phosphorylation sites in the GPCR C-tail (42). Supporting the significance of C-tail phosphorylation and β-arrestin in receptor trafficking, our phosphodeficient version of GIPR results in lower internalization (Fig. 5, *B–D*). In parallel, various GPCRs contain different types of PBM (I, II, and III) that we have demonstrated to bind with varying strengths to GIPC, in turn differentially stimulating myosin VI activity (8, 20). In this study, deletion of the PBM site (GIPRΔPBM) in GIPR leads to diminished receptor internalization (Fig. 5, *B–D*). Supporting the significance of the GIPR PBM, this variant is associated with a metabolic phenotype (43). Together, we propose that the diversity of PBM and phosphomotifs enable differential regulation of GPCR trafficking to tune the spatiotemporal signaling response.

## Experimental procedures

### Cell culture

HEK293T Flp-In T-Rex cells (referred to as HEK293 cells, ThermoFisher) and HEK293 β-arrestin half knockdown cells (referred to as HEK293 Δβ-arr) (22) were maintained in Dublecco’s modified eagle medium (DMEM, ThermoFisher), supplemented with GlutaMax (Thermo Fisher), 20 mM HEPES buffer (Corning) and 10% fetal bovine serum (FBS, GenClone). Cells were routinely cultured in 10 cm tissue-culture treated plates at 37 °C with 5% CO_2_.

INS-1832/3 cells (MilliporeSigma) were cultured in RPMI-1640 media, supplemented with 10% FBS, 10 mM HEPES, 2 mM L-Glutamine, 1 mM sodium pyruvate and 0.05 mM β-mercaptoethanol. *Spodoptera frugiperda* insect cells (referred to as Sf9 cells, Thermo Fisher) were maintained at 28 °C in Sf900-II media (ThermoFisher) supplemented with antibiotic-antimycotic (ThermoFisher).

### Plasmid construction

All plasmid constructs used in this study were generated using standard cloning procedures. Human V2R, β2AR, GIPR, GIPC and β-arrestin2 (β-arr2) were cloned into the pcDNA5/FRT vector backbone. All GIPR constructs in pcDNA contained FLAG and mNeonGreen (mNG) tags at the N-terminus of the GPCR. Last five residues (L462-C466) were deleted for generating GIPRΔPBM. Phosphorylation sites in the C-tail were mutated to glutamate or alanine for phosphomimetic or phosphodeficient versions of the receptor, respectively (refer to Figs. 5*A* and S7*A* for the C-tail sequences). For the colocalization experiment, tagRFP was included at C-terminus of GIPC and at N-terminus of β-arr2.

Constructs used for bimolecular FRET and motility assays contained a FLAG tag for purification and were cloned into pBiex backbone. For GIPR C-tail constructs used in the bimolecular FRET assay, residues V399-C466 were cloned with mCit tag at N-terminus. Phosphodeficient (pd) and phosphomimetic (pm) variants of GIPR C-tail were cloned by mutating the phosphorylation residues to alanine or glutamate, respectively (Fig. S9*A*). Full length myosin VI, GIPC-mCer-pBiex, and GIPC-pBiex were cloned as described previously (29).

### Internalization assay

HEK293 WT or Δβ-arr cells were seeded on poly-L-lysine coated glass coverslips in 35-mm plates (GenClone) at 30% density. The next day, cells were transfected with 4 μl polyethylenimine (PEI; PolySciences) and 1 μg of DNA of the indicated GIPR construct. For the co-localization experiment, 1 μg FLAG-mNG-GIPR and 0.5 μg of either GIPC-tagRFP or tagRFP-β-arr2 were transfected with 6 μl PEI. Internalization assay was carried out 24 h post-transfection for all conditions, except GIPR + β-arr2 co-localization, transfection was allowed for 48 h.

Cells were blocked in DMEM with 0.1% BSA for 15 min at 4 °C prior to labeling. Anti-FLAG antibody from Millipore Sigma or GenScript performed similarly for labeling surface receptors. Anti-FLAG antibody was incubated with a secondary AlexaFlour 546 or 647 (Thermo Fisher) in DMEM with 0.1% BSA at 4 °C for 60 min. Anti-FLAG + AlexaFlour complex was used to label surface receptors in HEK293 cells expressing GIPR at 4 °C for 60 min. Next, cells were stimulated with GIP (1 μM; GenScript) for 15 min. When using TIP, cells were pretreated with TIP (100 μM; 15 min; MilliporeSigma) prior to agonist stimulation and TIP was included during GIP stimulation. Cells were fixed in 4% formaldehyde for 10 min at room temperature. Coverslips were then washed in PBS and mounted on glass slide using Prolong Diamond Anti-Fade (ThermoFisher). Coverslips were sealed the next day with valap (vaseline/lanolin/paraffin) and imaged on a Nikon Eclipse Ti inverted epifluorescence microscope with 100X oil immersion objective (1.4 numerical aperture) equipped with Evolve EMCCD camera (Photometrics) or PCO.edge sCMOS camera using Nikon Elements software (Nikon). Z-stacks (0.6 μm step size) were acquired, and maximum intensity projection (MIP) were created using Fiji (ImageJ) (44). Number of particles and cytosol-to-surface intensity were determined using Fiji, as described previously (20). At least three biological replicates were carried out for each condition, and 10 to 20 cells were analyzed per each condition in each biological replicate.

### Live-cell imaging

HEK392 WT or Δβ-arr cells were cultured on 45-mm round coverslips in 60-mm plates. Next day, the cells were transfected with FLAG-mNG-GIPR (1 μg) and 4 μl PEI. After 24 h of expression, GIPR was labeled using the procedure described under ‘Internalization assay.’ For live-cell imaging, the coverslip was mounted on the FCS2 chamber (Bioptechs) to maintain temperature at 37 °C and the cells were imaged in buffer (Hank’s Buffered Saline Solution, 0.2% glucose, 100 μM ascorbic acid). Cells were stimulated with GIP (1 μM) through perfusion into the FCS2 chamber. Myosin VI activity was blocked with TIP treatment (100 μM) during stimulation. GIPR internalization was recorded over time on a Nikon Eclipse Ti inverted epifluorescence microscope using 60× (1.4 NA) at 1.5× magnification. Images were acquired using a PCO.edge sCMOS camera.

### cAMP assay

HEK293 WT or Δβ-arr cells were seeded in 6-well plate at 30% cell density and transfected with 1 μg FLAG-mNG-GIPR or β2AR-mCit using 4 μl PEI for 24 h. For cAMP in INS cells, untransfected cells were seeded in a 6-well plate and processed the next day. On the day of the assay, cells were harvested in culturing media and centrifuged at 350 g for 5 min at room temperature. Next, cells were resuspended in stimulation buffer (PBS, 0.2% glucose, 0.5 mM ascorbic acid), cell count was determined using a hemocytometer (Countess II) and added to an opaque 384-well plate (Greiner Bio-One) at 1.5∗10ˆ4 cells/well. The samples were then processed according to the manufacturer’s instructions for the cAMP glo assay kit (Promega). Cells were stimulated with 1 μM GIP ± 10 μM TIP for 15 min at room temperature or 37 °C, and lysis buffer (Promega) was added to stop the reaction. Cells were not treated with any PDE inhibitor. Absorbance was recorded on a plate reader (Tecan Spark) with 500 ms integration. Basal cAMP (no agonist) was subtracted from respective samples to calculate the amount of cAMP. Surface receptor expression levels in HEK293 WT or Δβ-arr were measured by recording fluorescence spectra on FluoroMax-4 spectrofluorometer (Horiba Scientific - excitation 470 nm, emission 495–600 nm, 4 nm bandpass). At least four technical replicates and three biological replicates were performed for each condition.

### ERK1/2 (ERK) assay

HEK293 WT or Δβ-arr cells were seeded in 35-mm plates at 30% confluency. The next day, media was exchanged to serum-free DMEM and cells were transfected with FLAG-mNG-GIPR (1 μg) using 4 μl PEI.

### pERK measurement—Revvity

To determine pERK levels with the Revvity kit, cells were harvested in serum-free DMEM 24 h after transfection and serum starvation. Cells were then added to a white 384-well plate (Greiner Bio-One) at 1∗10ˆ4 cells/well. The pERK assay was carried out following the manufacturer’s instructions (Revvity). Cell density was used as a measure to normalize across different conditions. Cells were incubated with 1 μM GIP for 15 min at room temperature or 37 °C. To block myosin VI activity, TIP was added at 10 μM along with the agonist GIP. Reactions were quenched by adding lysis buffer (Revvity), and the plate was incubated at room temperature for 30 min with shaking at 500 rpm. Pre-mixed antibody solution was added to each well and incubated at room temperature for 16 to 20 h. pERK levels were measured using HTRF-compatible plate reader (Molecular Devices - excitation 314 nm, cutoff 630 nm; emission 665 nm and 620 nm, cutoff 570 nm). pERK amount was calculated as the ratio between 665 nm and 620 nm values. At least four technical replicates and three biological replicates were included for each condition.

### pERK and total ERK (tERK) assay—Western blot

After 24 h of expression and serum starvation, cells were stimulated with buffer, GIP (1 μM) and/or TIP (10 μM) for 15 min at 37 °C. Next, cells were washed in ice-cold PBS and lysed with buffer (20 mM imidazole pH 7.5, 7% sucrose, 200 mM NaCl, 4 mM MgCl_2_, 0.5 mM EDTA, 1 mM EGTA, 5 mM DTT, 0.5% IGEPAL, 5 μg/ml aprotinin, 5 μg/ml leupeptin, 5 μg/ml PMSF). Lysates were centrifuged at 14,000*g* for 10 min at 4 °C. Laemmli SDS-loading buffer was added to the supernatant and samples were then subjected to gel electrophoresis on a 10% SDS-PAGE gel. The proteins were transferred to a nitrocellulose membrane at 300 mA for 180 min at 4 °C and blotted using anti-tERK (p44/42 ERK, Cell Signaling 4695S) or anti-pERK (phospho-p44/42 ERK, Cell Signaling 4376S) primary antibody and anti-rabbit secondary antibody. Immunoblots were developed for 2 minutes and the images were acquired on Licor imager. Band intensities were measured using Fiji (ImageJ) to determine levels of pERK and tERK. pERK levels were normalized by the amount of tERK in the respective sample. Three biological replicates were included for each condition.

### Glucose-stimulated insulin secretion (GSIS) assay

INS-1832/3 cells were seeded in 12-well plates at 1∗10ˆ6 cells/well density and processed for the GSIS assay at ∼90% confluency. Cells were washed in Krebs-Ringer bicarbonate buffer (KRB buffer; 130 mM NaCl, 5 mM KCl, 1.2 mM CaCl_2_, 1.2 mM MgCl_2_, 1.2 mM KH_2_PO_4_, 25 mM NaHCO_3_, 20 mM HEPES, 0.2% BSA), followed by glucose starvation in KRB buffer with 2.5 mM glucose for 2 hours at 37 °C. Next, cells were stimulated with GIP (1 μM) for 30 min at 37 °C at 2.5 mM glucose or 11 mM glucose. TIP was added at 10 μM to block myosin VI activity. Supernatant containing secreted insulin was collected and added to a 384-well plate. Insulin levels were measured according to the manufacturer’s instructions (Revvity) using an HTRF-compatible plate reader (Molecular Devices - excitation 314 nm, cutoff 630 nm; emission 665 nm and 620 nm, cutoff 570 nm). Briefly, antibody was added to the wells and incubated overnight before acquisition on the plate reader. Insulin concentration was calculated from an insulin standard curve. Insulin secretion at 2.5 mM glucose was used as the baseline for the respective conditions. At least four technical replicates and three biological replicates were included for every condition.

### Bimolecular FRET

Sf9 cells at 2∗10ˆ6 cells/ml density were transfected with DNA for 7 μg GIPC-mCer, 5 to 7 μg mCit-GIPR C-tail (WT, pm or pn) or 10 μg β-arr2 and Escort IV transfection reagent (MilliporeSigma). Proteins were purified from Sf9 cells 72-h post-transfection, as described previously (20). Briefly, cells were harvested and lysed in lysis buffer (20 mM imidazole pH 7.5, 7% sucrose, 200 mM NaCl, 4 mM MgCl_2_, 0.5 mM EDTA, 1 mM EGTA, 5 mM DTT, 0.5% IGEPAL, 5 μg/ml aprotinin, 5 μg/ml leupeptin, 5 μg/ml PMSF). The lysate was clarified by centrifugation at 176,000*g* for 25 min at 4 °C. Next, the supernatant was incubated anti-FLAG affinity resin (MilliporeSigma) for 60 min at 4 °C. Then, the resin was washed three times in wash buffer (20 mM imidazole pH 7.5, 7% sucrose, 150 mM KCl, 5 mM MgCl_2_, 1 mM EDTA, 1 mM EGTA, 5 mM DTT, 5 μg/ml aprotinin, 5 μg/ml leupeptin, 5 μg/ml PMSF). Elution of the bound protein was carried out by incubating the resin with FLAG peptide (GenScript) overnight at 4 °C. Verification and quantification of protein expression was done by running the purified protein samples on SDS-PAGE gel (Fig. S8).

FRET assays were performed with proteins diluted in FRET buffer (20 mM HEPES pH 7.4, 25 mM KCl, 5 mM MgCl_2_). GIPC-mCer was used at 30 nM and mCit-GPCR C-tails and β-arr2 were added at 300 nM. WT, pm or pn variants of the GIPR C-tail were used where indicated. Spectra for FRET samples were recorded on a FluoroMax-4 spectrofluorometer (Horiba Scientific) by exciting at 430 nm (8 nm bandpass) and emission from 450 nm to 650 nm (4 nm bandpass and 1 nm intervals). mCit-GPCR C-tail spectra ± β-arr2 were subtracted to account for cross-excitation. FRET ratio (525 nm/475 nm) was determined using peak intensity values for mCitrine (525 nm) and mCerulean (475 nm).

### Motility assay

Sf9 cells were transfected with DNA (7 μg myosin VI, 7 μg GIPC or 10 μg β-arr2) using Escort IV transfection reagent. Proteins were purified as described above. Purified β-arr2 was further desalted with a Zeba spin column (ThermoFisher), following the manufacturer’s protocol. GPCR C-tails (WT, pm or pn) were commercially synthesized (GenScript). Myosin VI motility assays were carried out as previously described (20). Briefly, flow chambers were created with collodion-coated coverslips attached to glass slides with strips of double-sided tape. Anti-His antibody (Qiagen, 0.02 mg/ml) in assay buffer (AB; 20 mM imidazole pH 7.5, 25 mM KCl, 4 mM MgCl_2_, 1 mM EGTA) was passed through the flow chamber and allowed to incubated for 4 min, followed by three washes with 10 μl AB-BSA (AB with 1 mg/ml BSA) and blocking with 10 μl AB-BSA for 2 min. Next, pre-mixed myosin VI (200 nM), GIPC (2 μM) and GIPR C-tail (100 μM) in AB-BSA were flown through the chamber and incubated for 4 min. For β-arr2 condition, β-arr2 (1 μM) in AB-BSA was flown separately through the chamber and incubated for 2 min. AB-BSA (10 μl) was used between each step to wash off unbound protein. Finally, actin motility mix (F-actin labeled with Alexa-647 phalloidin (ThermoFisher), 45 μg/ml catalase (EMD Millipore), 2 mM ATP, 1 mM phospho-creatine (MilliporeSigma), 0.1 mg/ml creatine phosphokinase (EMD Millipore), 0.6% glucose, 25 μg/ml glucose oxidase (EMD Millipore)), supplemented with GIPC and GIPR C-tail was flown through the flow chamber. A Nikon Eclipse Ti inverted epifluorescence microscope equipped with an oil immersion objective (100x, 1.4 NA) was used to record motility movies at 1 frame/sec for 3 min with PCO.edge sCMOS camera using Nikon Elements software (Nikon). Motility movies were analyzed using the MTrack plugin in Fiji. For each condition, 25 to 30 tracks were analyzed and all conditions were performed in three biological replicates.

### Colocalization analysis

A quantitative metric using signal to noise (S/N) ratio was developed to determine colocalization of GIPC or β-arr2 to GIPR using Fiji (ImageJ). The background for GIPC or β-arr2 channel was subtracted and the GIPR image was set to threshold to generate a mask. Both the images were multiplied to generate a results image containing the overlap of the two images to get a measure of GIPC/β-arr2 that colocalizes with the receptor (referred to as signal). The GIPR image was then inverted and multiplied to GIPC/β-arr2 to determine the amount that does not colocalize with the receptor (referred to as noise). Then, the ratio of signal to noise was calculated and used as a measure of colocalization, where S/N ratio above 1 indicates colocalization. At least 10 cells were analyzed per experimental condition for three biological replicates.

### Statistical analysis

GraphPad Prism (version 10) was used to generate graphs and compute statistical significance. All data are represented as mean ± SD, and details of the statistical tests performed are mentioned in the figure legends.

## Data availability

All data are contained in this manuscript and microscopy images are available upon request.

## Supporting information

This article contains supporting information.

## Conflict of interest

The authors declare the following financial interests/personal relationships which may be considered as potential competing interests: S. S. is co-founder of Oxbow Therapeutics, LLC.
