# Supplementary material for: β-arrestin and myosin VI cooperatively control glucose-dependent insulinotropic peptide receptor (GIPR) internalization and signaling dynamics
Source: J Biol Chem. 2026 Jul 16;302(9):113339. doi: 10.1016/j.jbc.2026.113339 (PMC13485621; doi:10.1016/j.jbc.2026.113339)
Supplement: Supplementary file 1 [file mmc1.pdf]

Supporting information

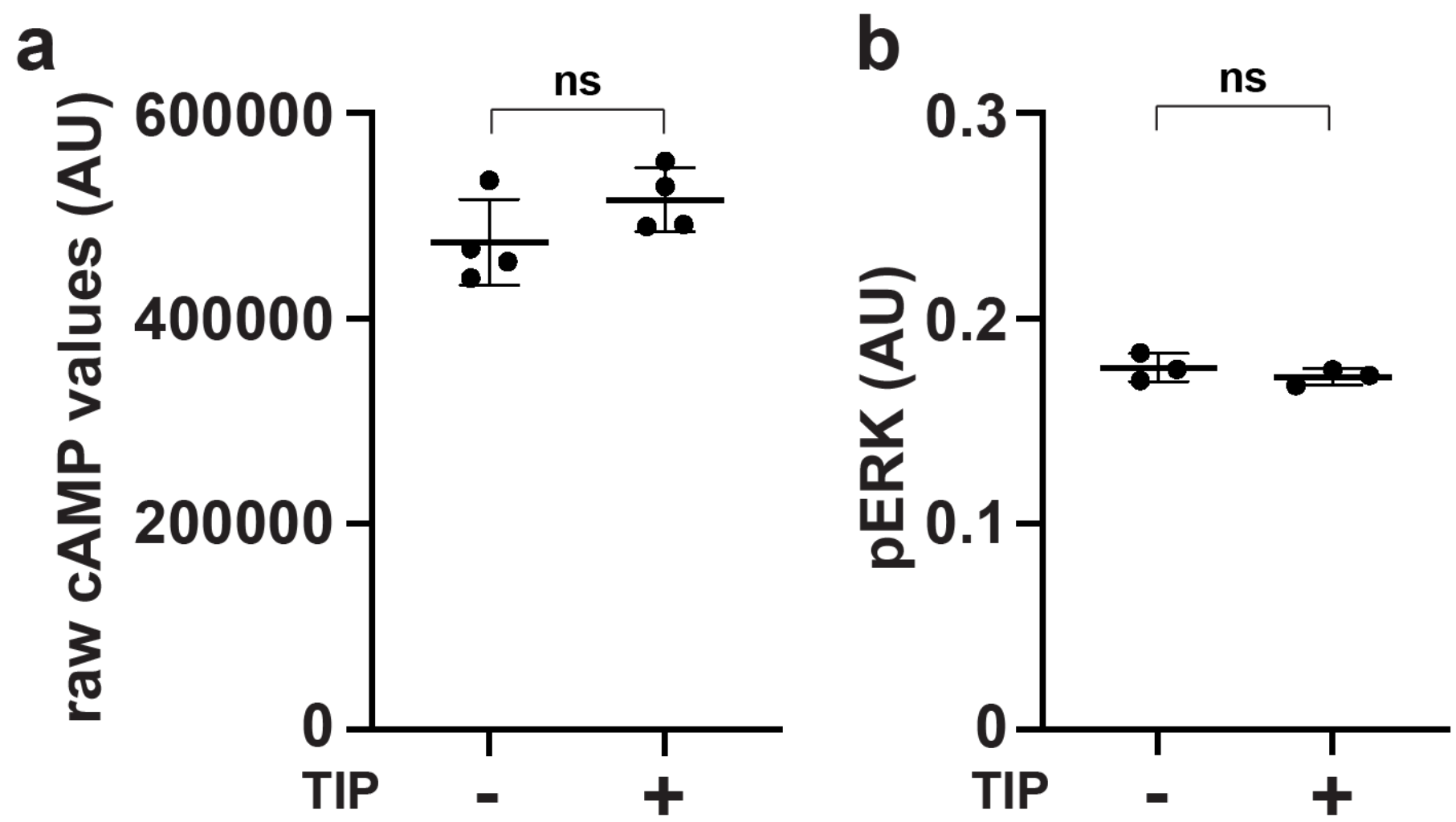

Figure S1: TIP does not affect basal cAMP and pERK output. (a) Raw luminescence values for HEK293 WT cells expressing GIPR, treated with TIP (10 µM) for 15 min at room temperature. cAMP is calculated by subtracting luminescence counts of treatment samples from buffer luminescence counts. (b) pERK levels at 0 min in response to buffer +/- TIP (10 µM) in HEK293 cells expressing GIPR. Mean +/- SD for at least three biological replicates. Unpaired t-test.

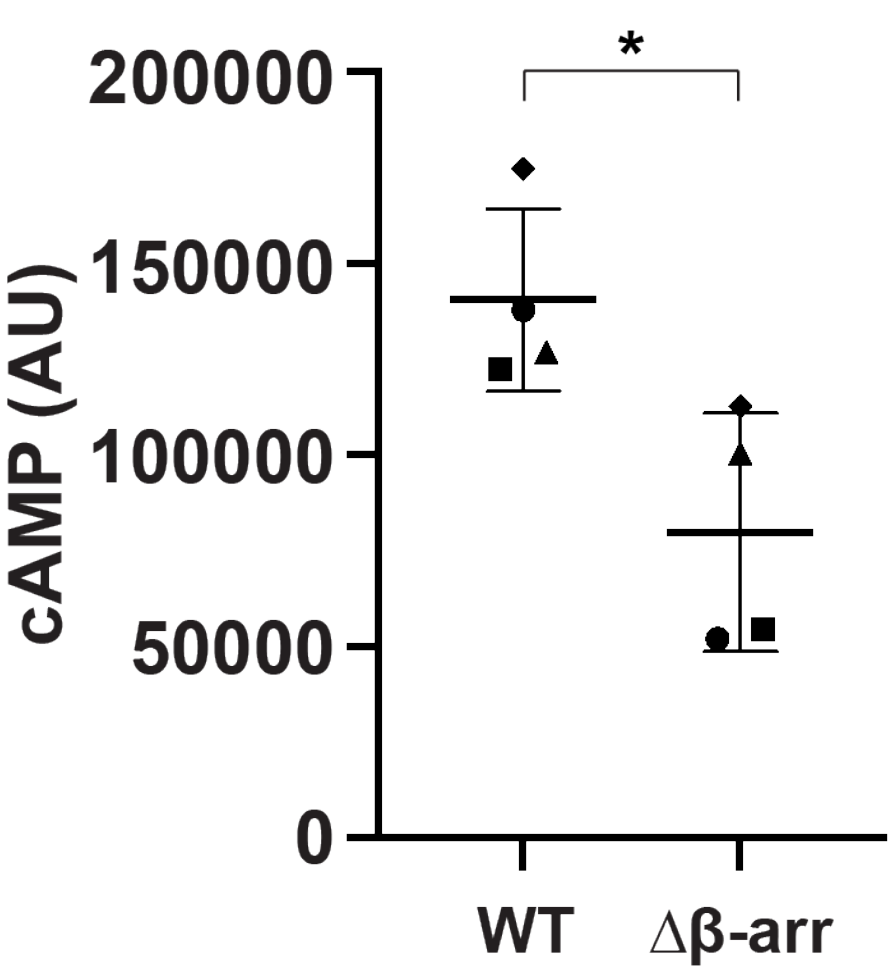

Figure S2: β2AR produces lower cAMP in Δβ-arr cells. cAMP production in HEK293 WT and Δβ-arr cells expressing β2AR, stimulated with isoproterenol (10 µM) for 10 min at room temperature. Mean +/- SD for four biological replicates. Unpaired t-test. *$p < 0.05$.

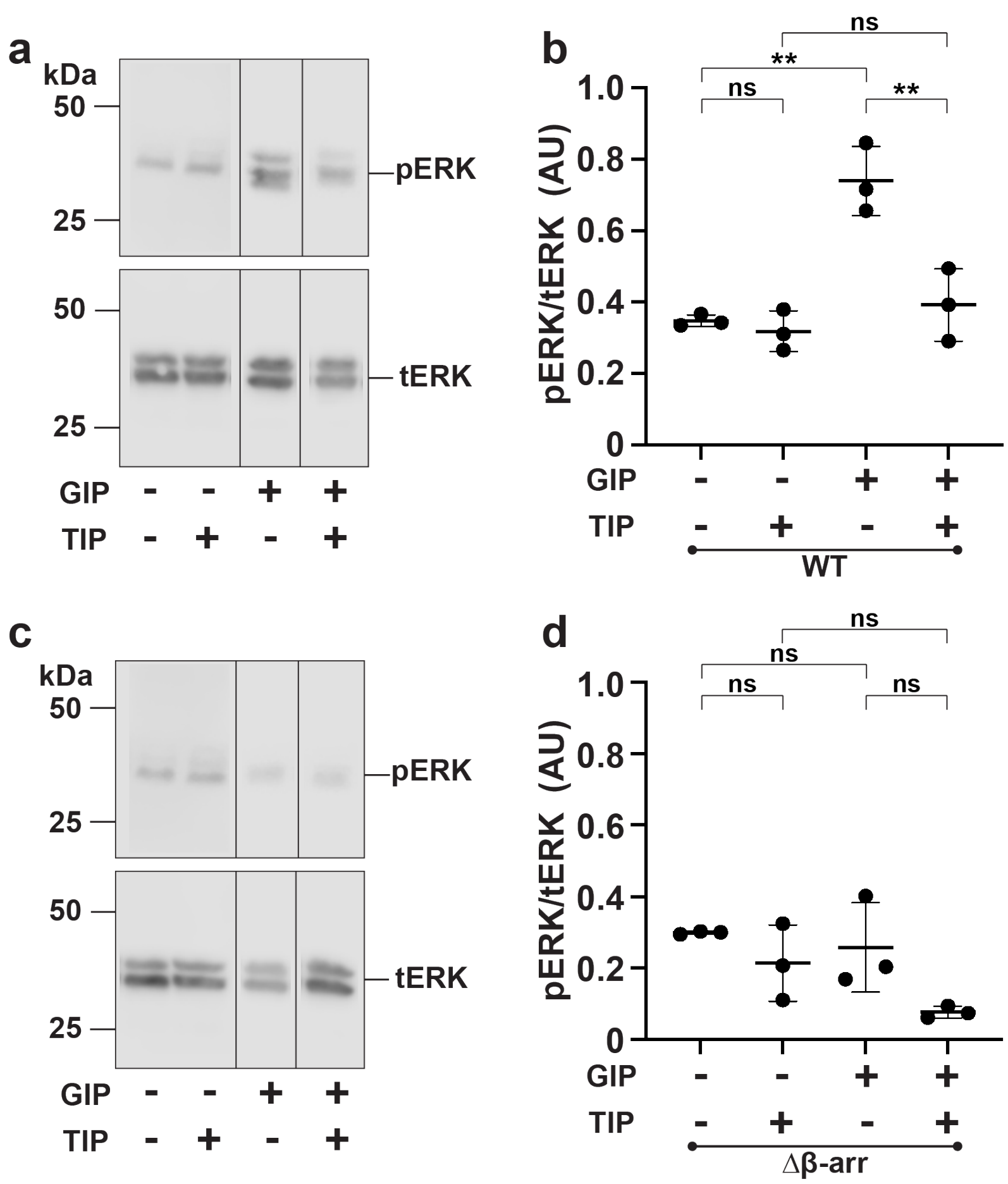

Figure S3: Normalization of pERK levels by total ERK (tERK) content. (a) Western blot for pERK and tERK levels in HEK293 WT cells expressing GIPR and treated with buffer +/- TIP (10 µM) and GIP (1 µM) +/- TIP (10 µM) for 15 min at 37°C. Representative lane is shown for each condition. (b) pERK levels in HEK293 WT cells normalized by tERK amounts, quantified from the blot. (c) Western blot for pERK and tERK levels in HEK293 Δβ-arr cells expressing GIPR for the same conditions shown for WT cells. Representative lane is shown for each condition. (d) Quantification of pERK in HEK293 Δβ-arr cells, normalized by tERK amounts. Mean +/- SD for three biological replicates. One-way ANOVA. $^{**}p < 0.01$.

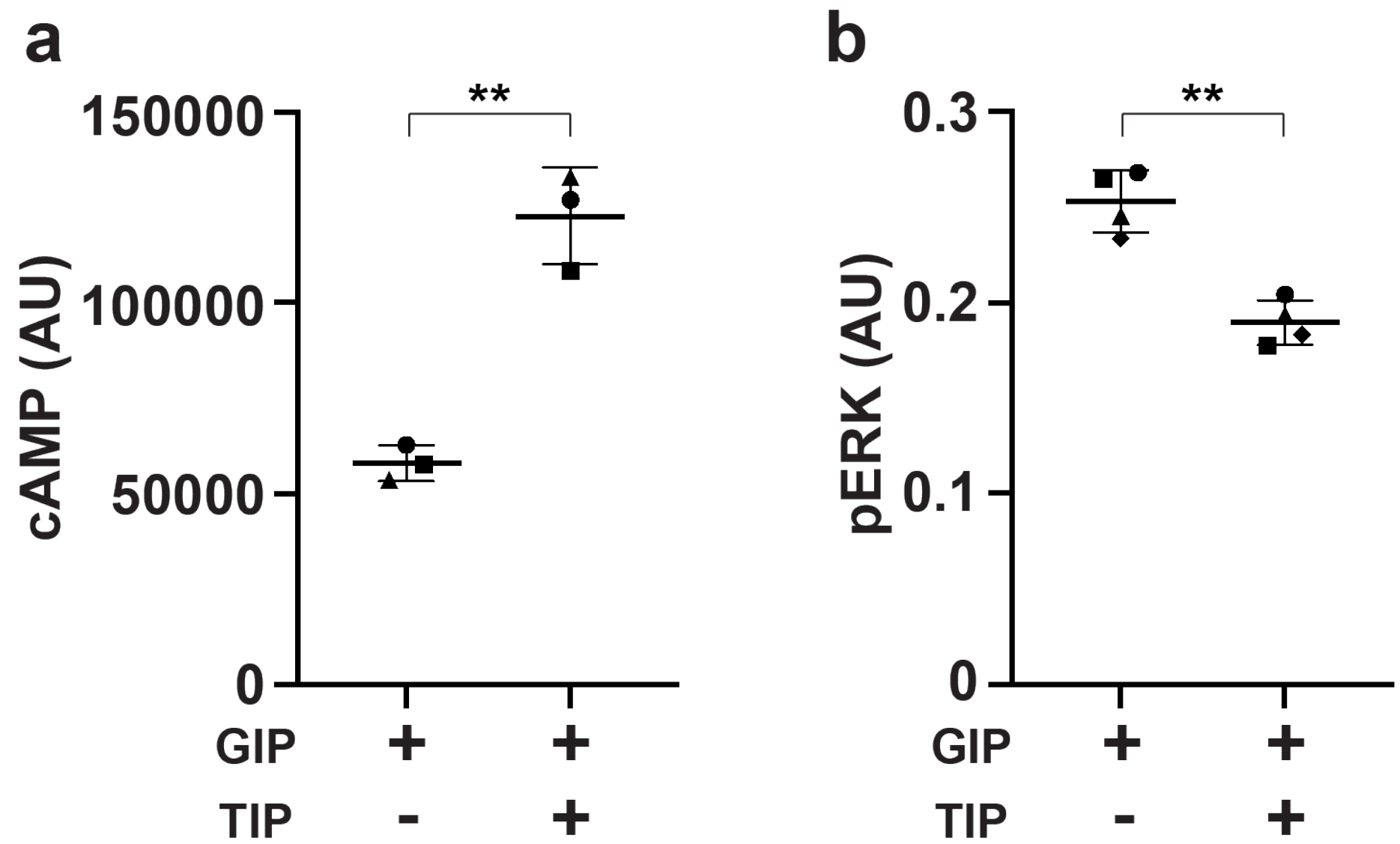

Figure S4: Effect of TIP on cAMP and pERK levels is preserved at 37°C. (a-b) cAMP (a) and pERK (b) levels in HEK293 WT cells expressing GIPR, stimulated with GIP (1 µM) +/- TIP (10 µM) for 15 min at 37°C. Mean +/- SD for three-to-four biological replicates. Unpaired t-test. **p < 0.01.

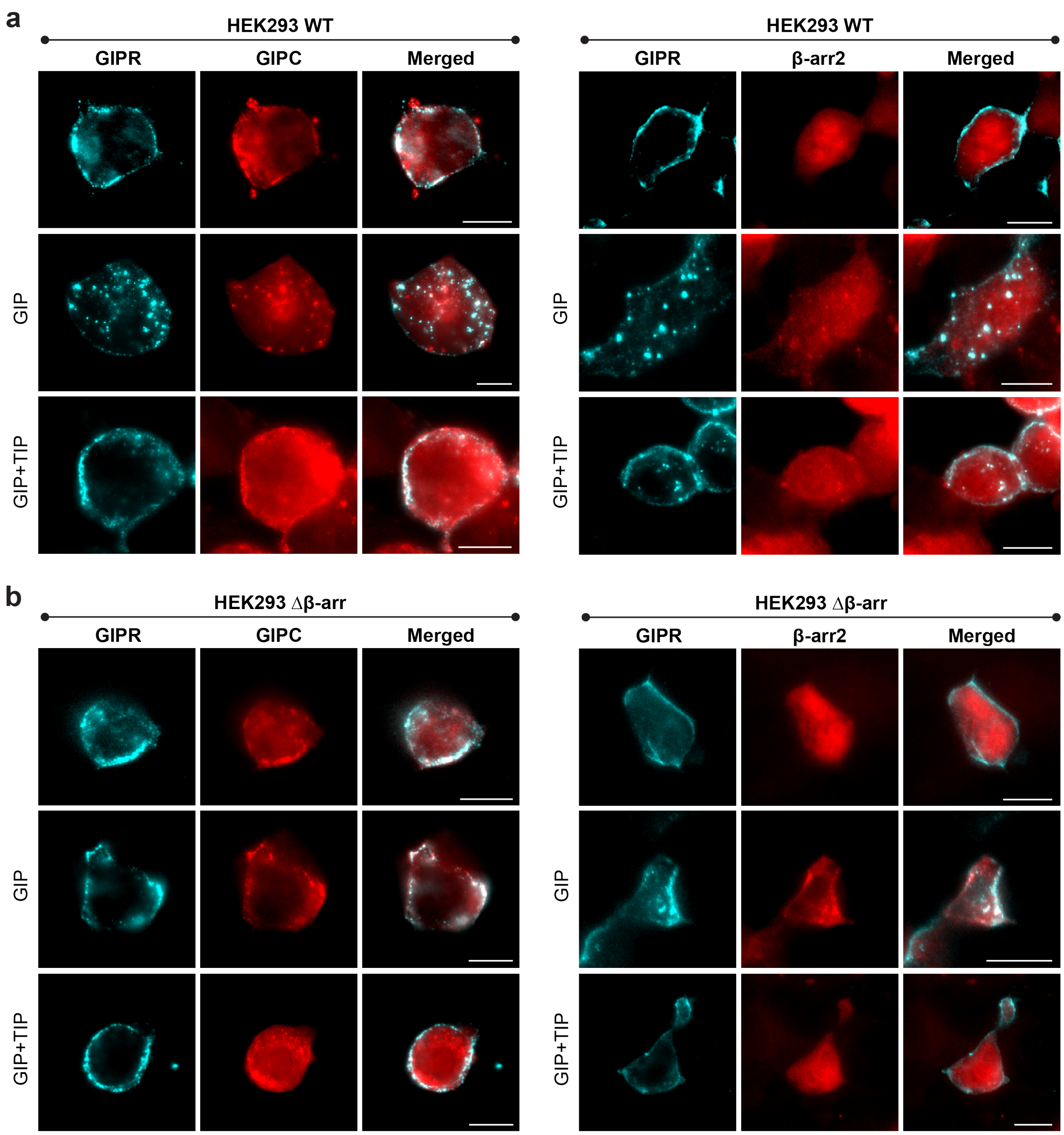

Figure S5: Colocalization of GIPC and β-arr2 with GIPR. (a-b) Co-expression of FLAG-GIPR with GIPC-TagRFP or TagRFP-β-arr2 in HEK293 WT (a) or Δβ-arr cells (b). Receptor was visualized by immunostaining with anti-FLAG antibody + secondary AlexaFlour 647. Images are shown for basal or GIP stimulation +/- TIP conditions at 37°C. Scale bar, 10 μm.

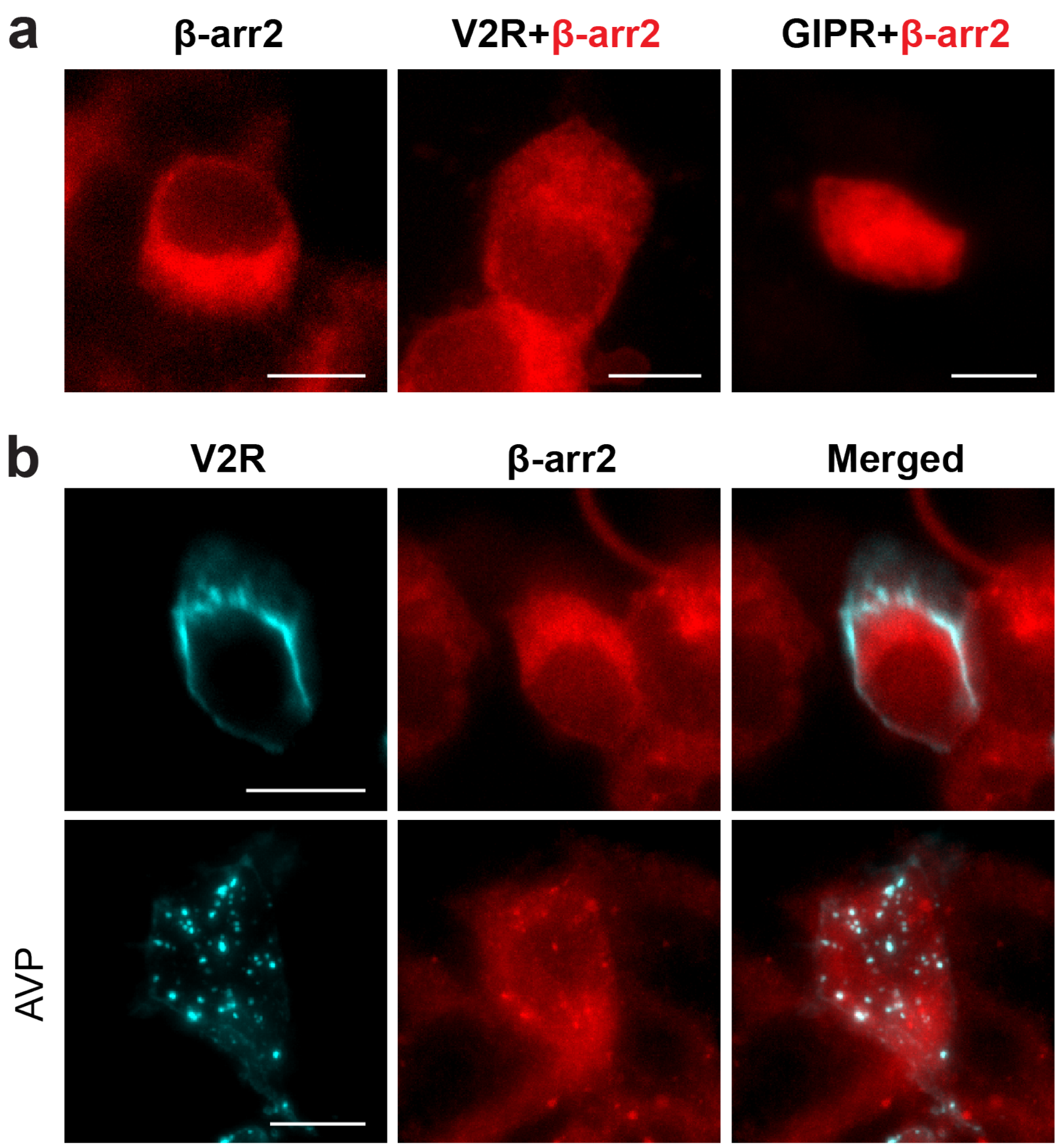

Figure S6: Translocation of β-arr2 to nucleus is GIPR-dependent. (a) HEK293 WT cells expressing tagRFP-β-arr2 alone or co-expression of β-arr2 with V2R or GIPR. Images are shown only for the β-arr2 channel for each condition. (b) Co-expression of V2R with β-arr2 in HEK293 WT cells. V2R was visualized by immunostaining with anti-FLAG antibody + secondary AlexaFlour 647. Images are shown for basal or AVP stimulation +/- TIP conditions at 37°C. Scale bar, 10 μm.

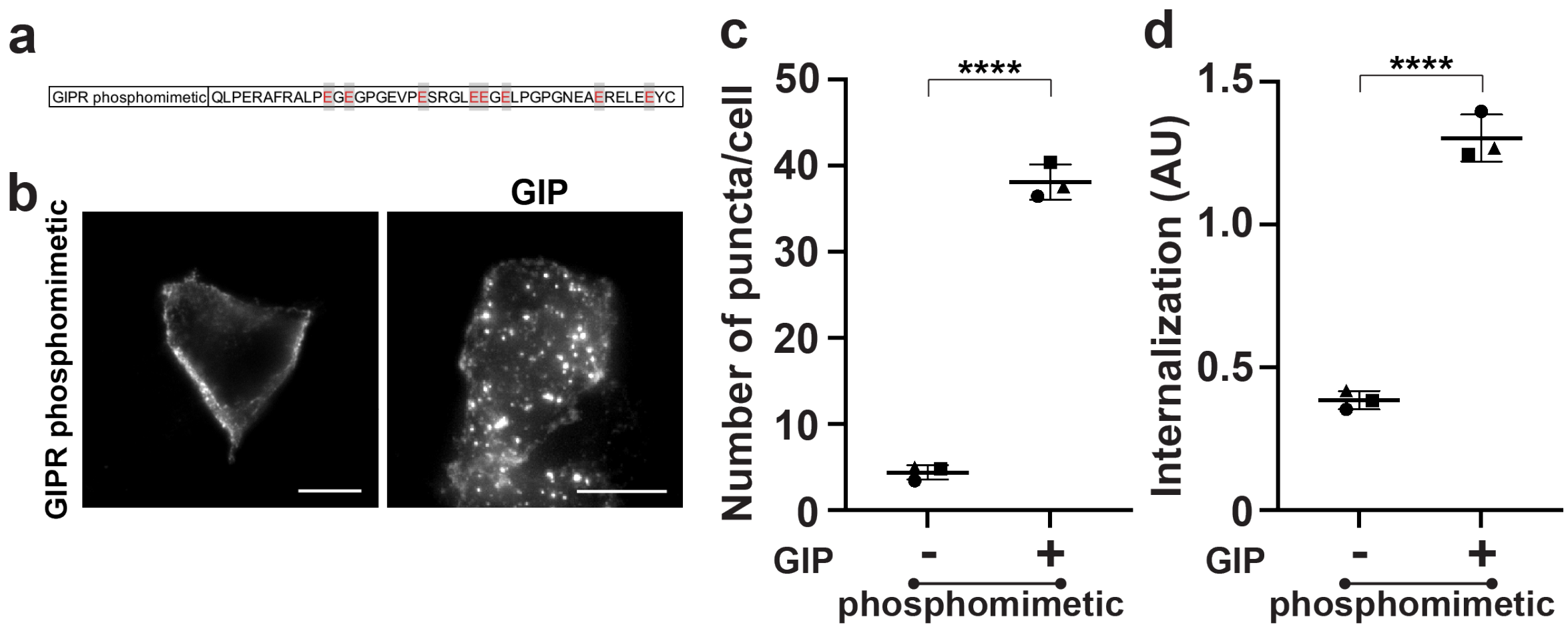

Figure S7: GIPR phosphomimetic undergoes complete internalization. (a) Schematic representation of GIPR phosphomimetic construct. Phosphorylation sites highlighted in gray were mutated to glutamate to generate phosphomimetic version of the receptor. (b) Representative images of HEK293 WT cells expressing GIPR phosphomimetic under basal and GIP stimulation at 37°C. Cells were incubated with anti-FLAG antibody and AlexaFlour 546/647 for surface labeling of the receptor. Scale bar, 10 μm. (c-d) Number of puncta/cell (c) and ratio for cytosol-to-cell membrane intensity (d) for GIPR phosphomimetic under basal and GIP-stimulated cells. Data is represented as mean +/- SD for three biological replicates. One-way ANOVA with Tukey's post hoc test. ****p < 0.0001.

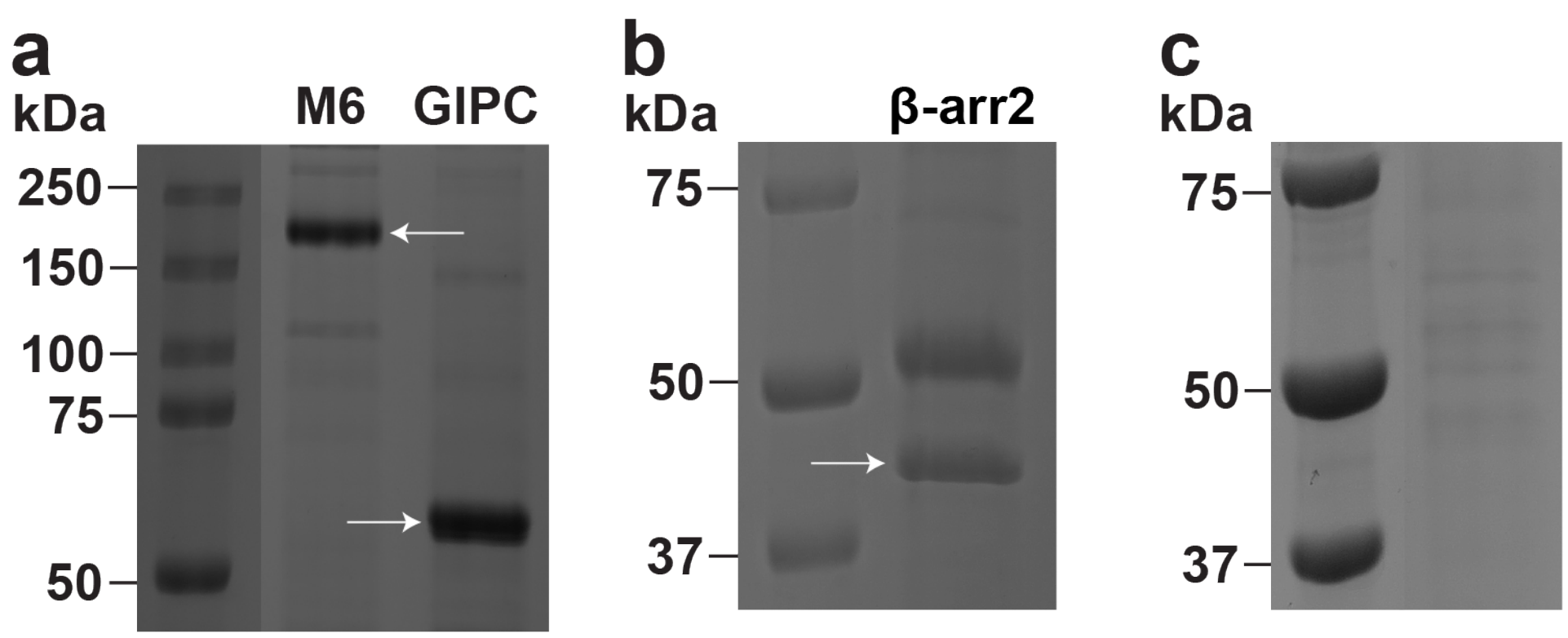

Figure S8: Purified proteins used in FRET and motility assays. (a-c) Coomassie staining of SDS-PAGE gel showing purified M6, GIPC (a), β-arr2 (b), and untransfected SF9 cells (c). White arrows indicate the expected band for respective protein. The additional band in β-arr2 sample is specific to β-arr2 (b), as untransfected SF9 cells do not contain that band (c).

a

GIPR CT	QLPERAFRALPSGSGPGEVPTSRGLSSGTLPGPGNEASRELESYC
GIPR CT pd	QLPERAFRALPAGAGPGEVPASRGLAAGALPGPGNEAARELEAYC
GIPR CT pm	QLPERAFRALPEGEGPGEVPESRGLEEGELPGPGNEAERELEEYC

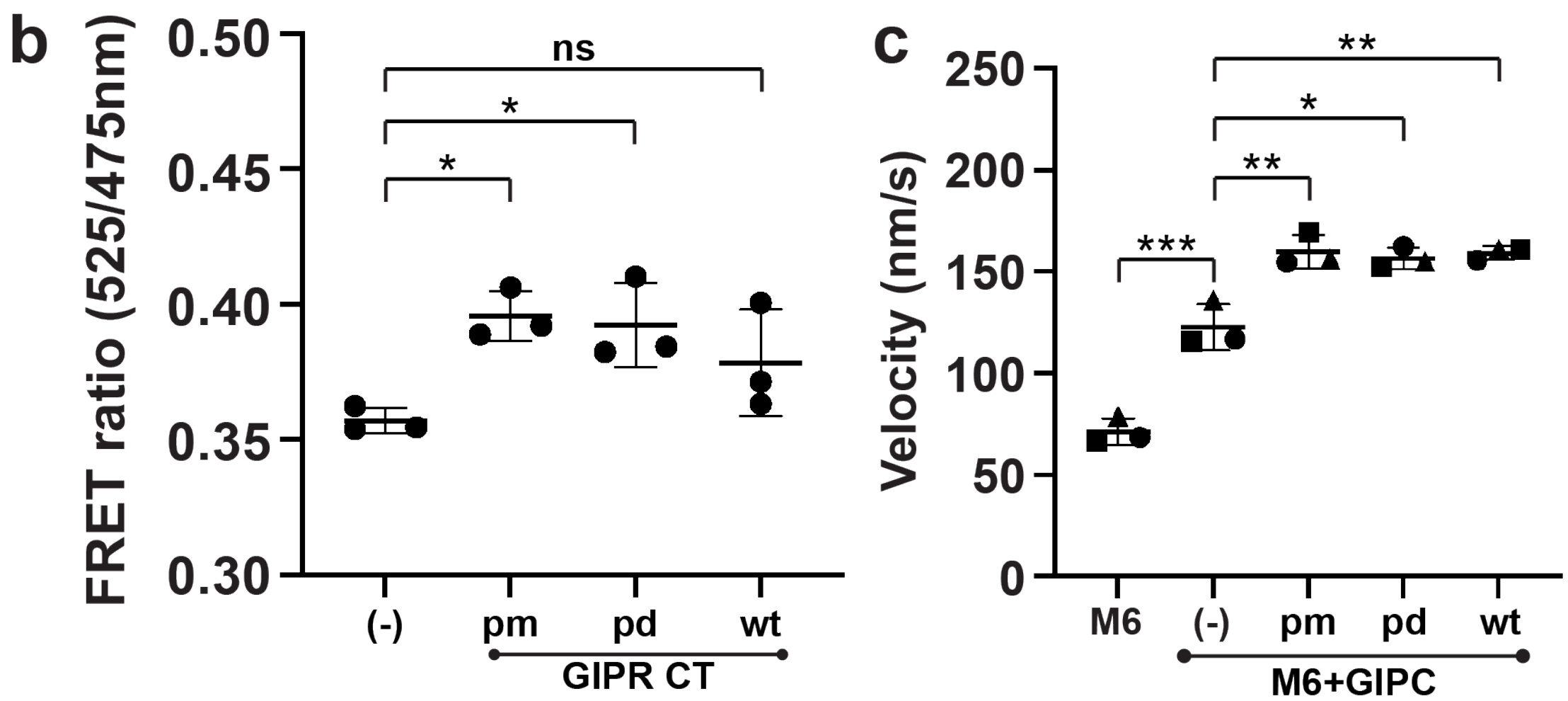

Figure S9: GIPR C-tail interacts with GIPC and activates myosin VI. (a) Sequence of GIPR CT (wild-type (wt), phoshonull (pn), and phoshphomimetic (pm)) used in the FRET and motility assays. (b) FRET ratio (525/475 nm) for bimolecular FRET between mCer-GIPC (30 nM) and mCit-GIPR C-tail (300 nM). Data is represented as mean +/- SD for three biological replicates. One-way ANOVA with Dunnett's post hoc test. *p < 0.05. (c) Myosin VI motility with GIPC and GIPR C-tail (wt, pn, or pm). Data is represented as mean +/- SD for three biological replicates. One-way ANOVA with Tukey's post hoc test. ***p < 0.001, **p < 0.01, *p < 0.05.

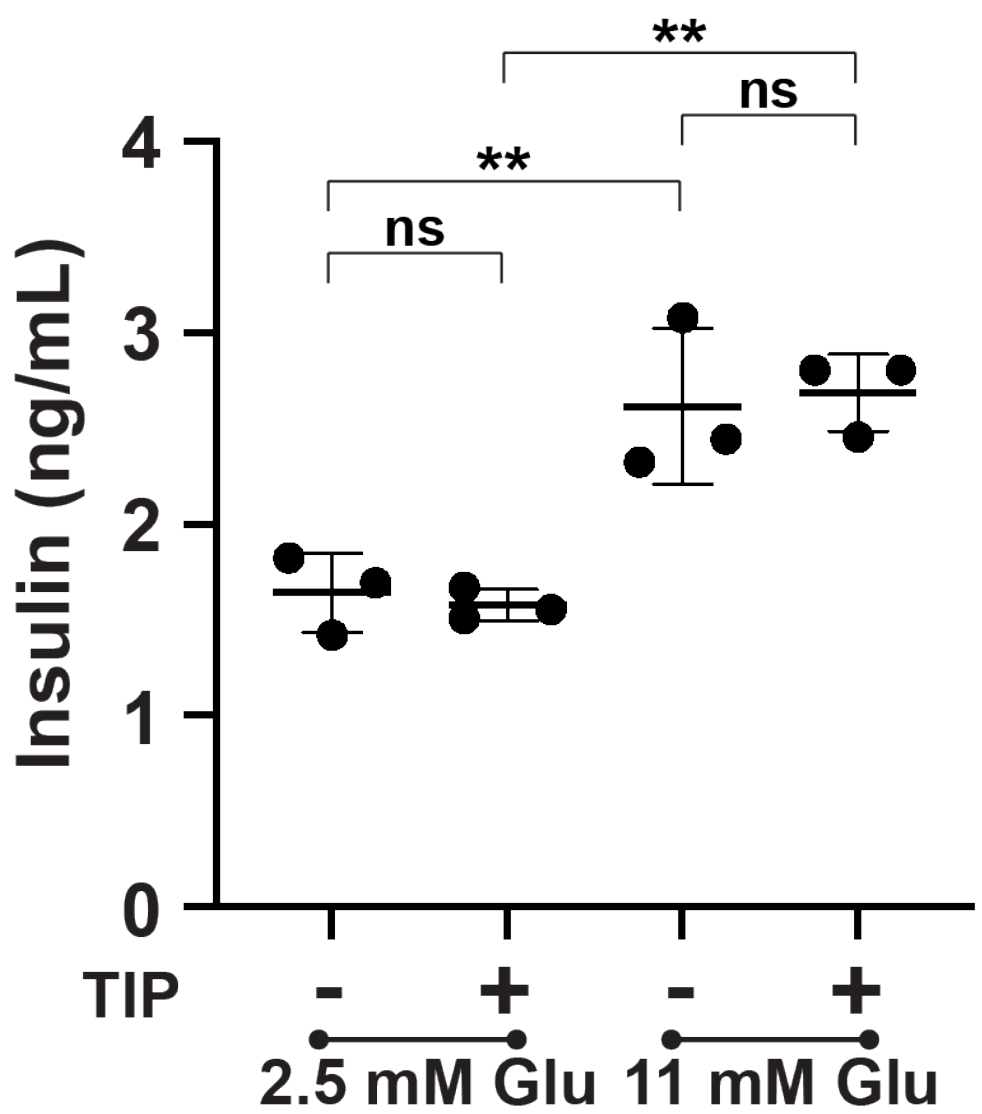

Figure S10: Glucose stimulated insulin secretion is not affected by TIP. Raw values for Insulin secretion in INS cells at 2.5 mM or 11 mM glucose for 30 min at 37°C. Cells were treated with TIP (10 μM). Data is represented as mean +/- SD for three biological replicates. Two-way ANOVA with Sidak's post hoc test. **p < 0.005.

Table S1: One-way ANOVA with Dunnett's post hoc test for FRET experiment between GIPC and GIPR CT pm, GIPR CT pd, and GIPR CT wt.

Comparison	Mean Difference	p-value	Summary
GIPC vs GIPR CT pm	-0.03878	0.0200	*
GIPC vs GIPR CT pd	-0.03537	0.0312	*
GIPC vs GIPR CT wt	-0.02147	0.1985	ns

Table S2: One-way ANOVA with Tukey's post hoc test for motility assay for M6 and GIPC, GIPR CT.

Comparison	Mean Difference	p-value	Summary
M6 vs. M6+GIPC	-51.50	***	0.0006
M6 vs. M6+GIPC+GIPR	-87.94	****	<0.0001
M6 vs. M6+GIPC+GIPR pd	-85.30	****	<0.0001
M6 vs. M6+GIPC+GIPR pm	-88.68	****	<0.0001
M6+GIPC vs. M6+GIPC+GIPR	-36.43	**	0.0098
M6+GIPC vs. M6+GIPC+GIPR pd	-33.80	*	0.0166
M6+GIPC vs. M6+GIPC+GIPR pm	-37.17	**	0.0085
M6+GIPC+GIPR vs. M6+GIPC+GIPR pd	2.635	ns	0.9995
M6+GIPC+GIPR vs. M6+GIPC+GIPR pm	-0.7402	ns	>0.9999
M6+GIPC+GIPR pn vs. M6+GIPC+GIPR pm	-3.375	ns	0.9983
